# Unusual Clinical Presentation of Cutaneous Angiosarcoma Masquerading as Eczema: A Case Report and Review of the Literature

**DOI:** 10.1155/2013/906426

**Published:** 2013-10-07

**Authors:** Nhat Q. Trinh, Issra Rashed, Kelli A. Hutchens, Aileen Go, Edward Melian, Rebecca Tung

**Affiliations:** ^1^Stritch School of Medicine, Loyola University Chicago, Maywood, IL 60153, USA; ^2^Department of Pathology, Loyola University Medical Center, Maywood, IL 60153, USA; ^3^Department of Hematology and Oncology, Loyola University Medical Center, Maywood, IL 60153, USA; ^4^Department of Radiation Oncology, Loyola University Medical Center, Maywood, IL 60153, USA; ^5^Department of Dermatology, Loyola University Medical Center, Maywood, IL 60153, USA

## Abstract

An unusual case of cutaneous angiosarcoma clinically mimicking eczema is described. A 98-year-old Caucasian male presented with a 6-month history of a flesh-colored, subcutaneous nodule on his left forehead with contralateral facial erythema and scaling that had been previously diagnosed as eczema. Despite treatments with topical steroids and moisturizers, the condition did not resolve. At our clinic, excisional biopsy of the forehead lesion and scouting biopsies from the contralateral cheek were performed which revealed cutaneous angiosarcoma. The described case illustrates that dermatitis-like features should be considered as a rare clinical manifestation of cutaneous angiosarcoma. It also demonstrates that these lesions may respond well to radiotherapy as a single modality.

## 1. Introduction

Angiosarcoma is a highly invasive tumor of vascular endothelial origin with poor prognosis and high rates of recurrences; therefore, early detection is essential to its treatment. The following case is reported because of the unusual clinical presentation of the malignant tumor in an elderly patient.

## 2. Case Report

In early 2012, a 98-year-old robust Caucasian male presented to our clinic with a 6-month history of an enlarging flesh-colored nodule on his forehead. Following the development of this lesion, mildly pruritic red, scaly patches appeared on the right side of his face. A clinical diagnosis of eczema was rendered by an outside physician. For his presumed dermatitis, a course of therapy including topical hydrocortisone and moisturizers was prescribed. While he complied with the outlined regimen, his symptoms did not improve.

**Figure 1:**
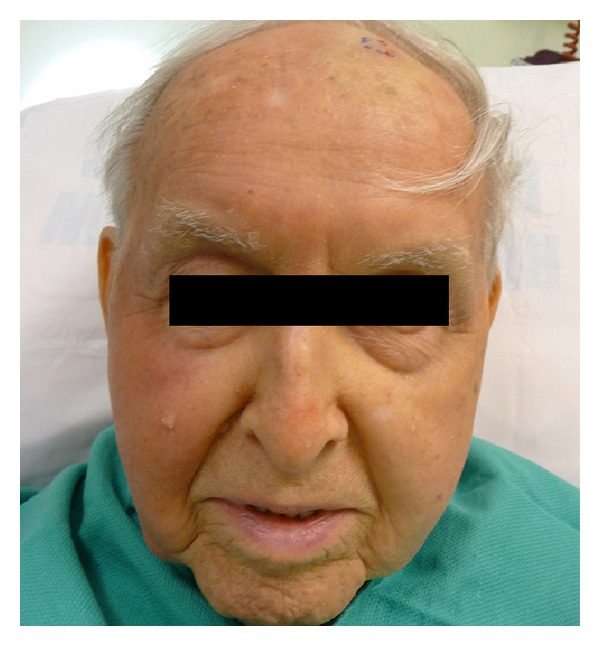
Clinical presentation of the patient with angiosarcoma prior to treatment.

**Figure 2:**
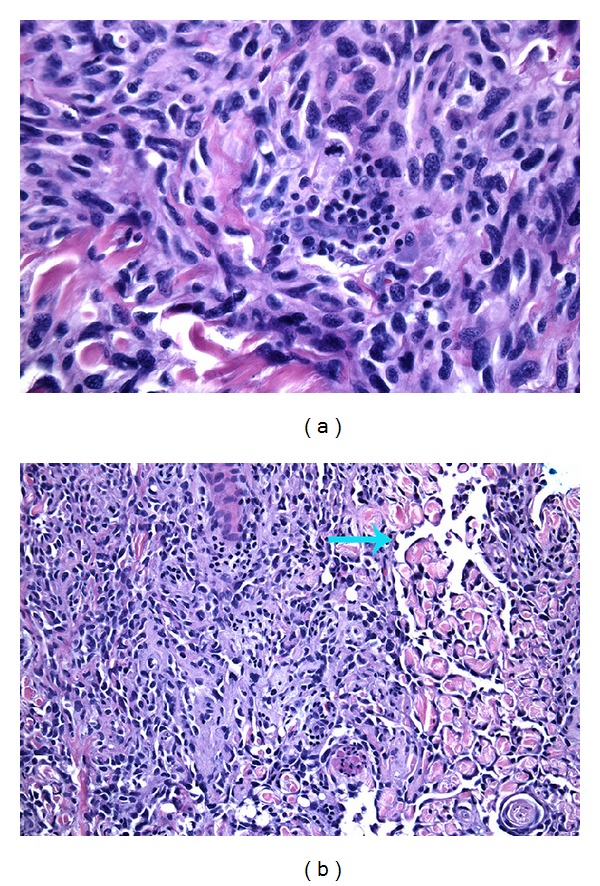
The main tumor was bulky and composed of atypical and pleomorphic spindle cells with numerous mitotic figures (a). On the periphery of the tumor, areas of vessel formation with hyperchromatic and “hob-nailing” (blue arrow) of the neoplastic endothelium were seen (b).

On clinical examination, the patient was found to have a flesh-colored subcutaneous nodule measuring 1.5 × 0.9 cm in diameter on his left forehead and poorly demarcated red, scaly patches which were most prominent on the right contralateral cheek (Figure 1). An excisional biopsy was performed, which revealed angiosarcoma with positive lateral and deep margins (Figure 2). Immunohistochemical stains showed that the tumor cells were positive for CD34 and CD31 and negative for S100, LCA, CK7, and CK20. A wider local excision was attempted; however, we were not able to achieve clear margins. The majority of the wound was closed primarily, but the central area was left to heal secondarily.

**Figure 3:**
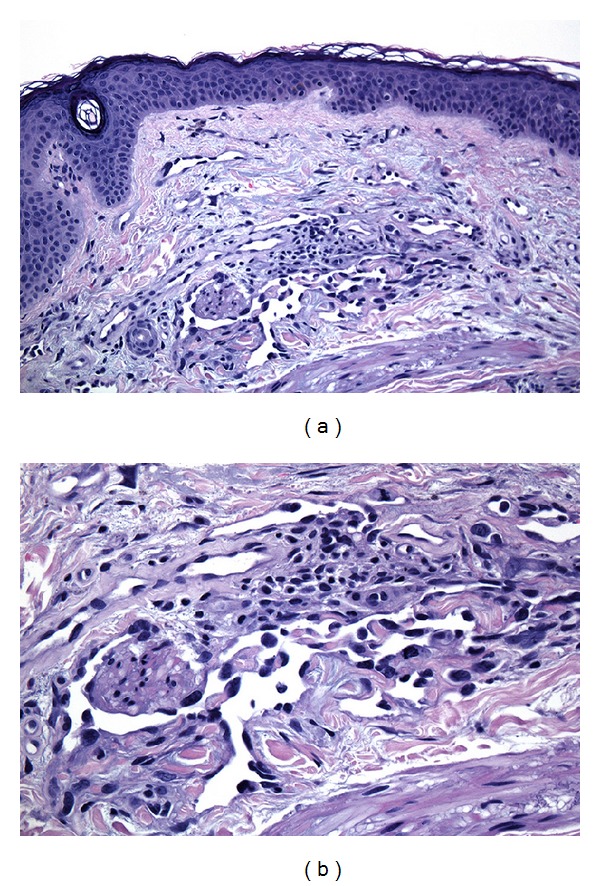
Scouting biopsies revealed a subtle infiltration in the superficial dermis with poorly formed malignant vascular structures similar to those seen at the periphery of the main tumor.

Because of the pervasive nature of this tumor, two scouting biopsies were taken from the right cheek, which were also consistent with angiosarcoma (Figure 3). Results of head/neck and chest noncontrast CT imaging were unremarkable for lymphadenopathy or metastatic spread.

Given the extensive involvement of the tumor in the scalp and face, complete resection was not feasible, and the patient was referred for evaluation by oncology and radiation oncology. The oncologist did not recommend administration of systemic therapy due to the patient's history of resected renal cell carcinoma, chronic renal failure with baseline creatinine >2 mg/dL, and cardiovascular disease significant for placement of multiple stents. After this multidisciplinary review, radiotherapy was recommended to treat the facial and scalp lesions. While the patient was waiting to begin radiation, one treatment of modified laser photodynamic therapy was administered but did not show any significant effect.

**Figure 4:**
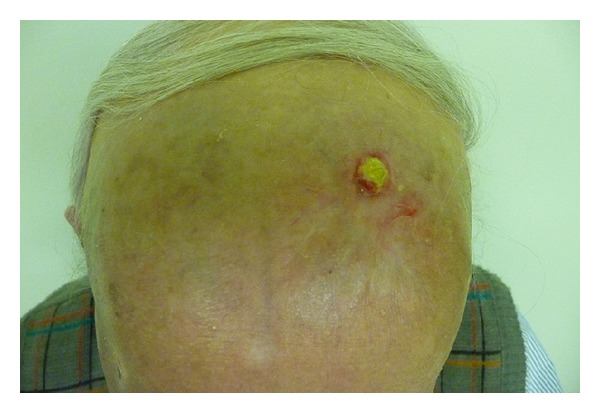
A localized infection of *Enterobacter cloacae* developed on the patient's left forehead.

**Figure 5:**
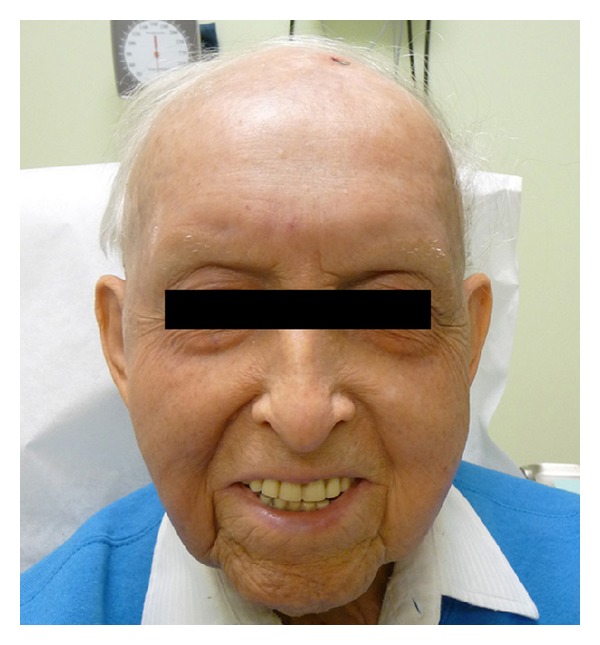
Presentation of the patient 1 month after treatment with radiation treatment as a single modality.

The scalp, face, and bilateral cheeks were irradiated with thirty fractions of 2 Gy each for a total dose of 60 Gy. Toxicity was minimized by using 4 enface electron beams on a staggered start schedule. A 1 cm bolus was used to assure full dose to the skin surface. Scleral shields were used beneath the eyelids to protect the lenses and cornea. His treatment course was complicated by an episode of epistaxis, which exacerbated his underlying anemia of chronic renal disease necessitating hospitalization and blood transfusion. During his last month of treatment, the patient developed a localized infection of *Enterobacter cloacae* on the left forehead, which was resolved with topical garamycin (Figure 4). Results of follow-up physical examination by the multidisciplinary team at six months did not show any evidence of tumor recurrence (Figure 5). In-field biopsies within the irradiated region were done 1 month after treatment confirming a complete pathologic response to the radiotherapy.

## 3. Discussion

Angiosarcoma is an aggressive malignant tumor of vascular endothelial origin that comprises approximately 2% of soft tissue sarcomas [1]. Due to the wide range of clinical scenarios that may be associated with this tumor, several broad categories have been designated: lymphedema-associated angiosarcoma, radiation-induced angiosarcoma, primary-breast angiosarcoma, angiosarcoma of the soft tissue, and cutaneous angiosarcoma (CA) [1]. Although primary local lesions of the disease can be effectively excised by surgical means, the high rate of local recurrences and distant metastases has a negative impact on patient survival.

Predisposing factors for angiosarcoma include trauma, chronic lymphedema, irradiation, and age (more common in the elderly population) [2, 3]. In many cases, however, the etiology remains to be properly elucidated [2].

While angiosarcoma may occur virtually anywhere in the body, it commonly presents on the head and neck. Retrospective analysis of a series of 47 patients with CA without lymphedema revealed that lesions most commonly presented on the scalp (49%) followed by the cheek (19%) [3]. Altogether, 95% of the cases had local lesions on the head and neck. In the same study, the majority of the patients (76%) were noted to be elderly men (59–92 years old) [3]. This predilection of CA in elderly males has been noted extensively in the literature [4–6]. 

In the clinical setting, the appearance of CA can be variable. Typical manifestations of CA have been described as raised purplish-red papules [6], rosacea-like lesions [7], and bruise-like lesions [2]. Due to the variability in the appearance of CA, the correct diagnosis can often be severely delayed. CA can present in an assortment of clinical guises that include, but are not limited to, Kaposi sarcoma [8], scarring alopecia [9], sebaceous cysts [10], and rhinophyma [11]. To date, we believe this is the first reported case of angiosarcoma mimicking eczema in the literature. 

Under the microscope, the most common histological patterns include atypical and pleomorphic (rounded, polygonal, or fusiform) endothelial cells exhibiting a diffuse epithelioid or spindle cell proliferation [3, 6, 10]. Immunohistochemical markers include von Willebrand factor, CD34, CD31, Ulex europaeus agglutinin 1, vascular endothelial growth factor (VEGF), and factor VIII antigen [2].

Prognosis of CA is poor with a reported 5-year survival rate ranging from 12 to 34% [3, 12, 13]. Depending on the modality of treatment, local recurrences have been observed in 35% to 86% of cases [5, 14]. In a retrospective case series of 47 patients with CA, all patients who developed metastases succumbed to the disease [3]. In another series of 48 patients with CA, 45 patients (93.8%) had disease recurrences (3 of which were local recurrences) [5]. In the same series, thirty-seven of those patients had distant metastases to the lungs and a median survival time of 4 months. The proclivity for distant pulmonary metastases has been well documented [15, 16]. Of note, angiosarcoma rarely metastasizes to the heart and major blood vessels [1, 3].

While the prognosis of patients with metastatic CA is significantly worse, the influence of other prognostic factors such as histological grade and size of the tumor is less clear. In one multivariate analysis, high mitotic counts were associated with worse outcomes [17]. In other studies, histological grade was the only significant prognostic factor [12, 18]. However, some investigators found tumor size to be a reliable prognostic tool: patients with tumors larger than 10 cm had almost 100% mortality rate [19, 20], while smaller tumors (<5 cm) correlated with better outcomes [4, 10].

Given the rarity of angiosarcoma, the optimal treatment has yet to be delineated. Current guidelines are mainly based on retrospective data. Single therapeutic modality (surgery, radiotherapy, or chemotherapy) is frequently employed; however, the high frequency of local recurrence has been disappointing [5, 12–14, 21]. As previously mentioned, complete eradication of the neoplasm is optimal when possible due to its high rate of local recurrences and distant metastases. The search for an effective systemic treatment for CA is ongoing, but several recent studies of primary tumors have reported success with a combined-modality approach of surgical resection followed by postoperative radiation therapy [4, 18, 20, 22]. 

Although the combination of surgery and radiotherapy has been effective in local control of CA, patients are still at risk for the development of distant metastases [4, 5]. This is in part due to treatment challenges involving the extensive and rapid spread of the disease. In addition, the original perceived size of the neoplasm may not correlate with the amount of microscopic tissue invasion [7]. Furthermore, surgical excision may not be a feasible option in some incidences, since resectable CA lesions constitute only a portion of the total cases.

For unresectable and regionally metastatic tumors, radiotherapy is a rational therapeutic approach due to the wide regional spread that can be treated while sparing the underlying tissue. Radiotherapy appears to improve local control and possibly overall survival based on retrospective series [5, 14, 23]. When using radiotherapy, an underestimation of the diffuse peripheral expanse of the tumor can limit the likelihood of treatment success [13, 21, 24]. Care must be taken to achieve full dose to the lesion surface and to protect the lenses and corneas as noted in our case. Employing a wide radiation treatment field is recommended [13, 21]. To the best of our knowledge, radiotherapy employed as a single modality treatment that resulted in complete remission of CA has been reported in only two cases [25, 26].

Due to the invasive nature of CA, another therapeutic option is chemotherapy. Doxorubicin-based chemotherapy remains the treatment of choice in metastatic soft tissue sarcomas. However, it is becoming clear that histologic subtypes differ in their susceptibility to chemotherapy and that treatment strategies should therefore be tailored according to histologic subtypes. Angiosarcomas appear to be particularly responsive to taxanes [27, 28], likely due to their antiangiogenic properties [6]. Dramatic and promising responses to agents like bevacizumab [29], sunitinib [30], and sorafenib [31] have also been reported, and their efficacy may be linked to the enhanced VEGF production in most cases of angiosarcoma [12, 32]. Although single agent therapy with these agents is tolerable, toxicity is not insignificant and patients with advanced age and significant clinical comorbidities may not qualify for therapy. Systemic chemotherapy in angiosarcoma is limited to patients who are not amenable to radiotherapy or curative-intent surgery. 

Recently, photodynamic therapy (PDT) has been suggested as an alternative modality for the treatment of CA. The photosensitizer (5-amino levulinic acid) used in this therapy becomes activated when photoirradiated by the proper wavelength. This technology has been shown to be effective for the control of primary CA tumors and the spontaneous remission of untreated metastases [33]. In the mouse model, PDT not only provoked cell death of angiosarcoma, but it also inhibited the proliferation potential of surviving tumors [34]. Additionally, investigators noted an increased migration of macrophages and other immune response cells at treated sites [34]. Further investigation will be necessary to delineate the molecular underpinnings and efficacy of this modality.

## 4. Conclusion

Cutaneous angiosarcoma is a very aggressive tumor with poor prognosis and high rates of recurrences; therefore, early detection is essential to its treatment (Table 1). Optimal therapy in our patient proved to be challenging owing to the presence of multiple significant comorbidities and the unusual and highly deceptive clinical presentation of the tumor. The case described herein illustrates that eczema-like features should be included as an atypical manifestation of cutaneous angiosarcoma. 

## Figures and Tables

**Table 1:** Overview of cutaneous angiosarcoma.

Location	Commonly found on the head and neck Predominately the scalp and cheek region	

Age	Generally occurs in the elderly population	

Sex	More common in males	

Poor prognostic factors	Presence of metastasis High mitotic count	Size of lesion >5 cm High histological grade

Typical appearances	Raised purplish-red papules Bruise-like lesions	Rosacea-like lesions

Differential diagnosis	Rhinophyma Sebaceous cysts Eczema	Kaposi sarcoma Scarring alopecia

Treatment options	*Surgical excision *	
	Recommend excision with wide negative margins or with postoperative radiotherapy	
	Caveat: lesions >5 cm are difficult to completely resect	
	*Radiotherapy *	
	Recommend wide treatment fields with doses of >50 Gy	
	Caveat: underestimation of the margins of tumor growth	
	*Chemotherapy *	
	Promising results shown with taxanes, bevacizumab, sunitinib, and sorafenib	
	Caveat: toxicity levels	
	*Photodynamic therapy *	
	Further research required to characterize the molecular mechanisms and optimize administration of this therapy	
	Caveat: depth of penetration	

## References

[1] Weiss SW, Goldblum JR, Folpe AL (2007). *Enzinger and Weiss's Soft Tissue Tumors*.

[2] Selim A, Khachemoune A, Lockshin NA (2005). Angiosarcoma: a case report and review of the literature. *Cutis*.

[3] Morgan MB, Swann M, Somach S, Eng W, Smoller B (2004). Cutaneous angiosarcoma: a case series with prognostic correlation. *Journal of the American Academy of Dermatology*.

[4] Guadagnolo BA, Zagars GK, Araujo D, Ravi V, Shellenberger TD, Sturgis EM (2011). Outcomes after definitive treatment for cutaneous angiosarcoma of the face and scalp. *Head and Neck*.

[5] Ogawa K, Takahashi K, Asato Y (2012). Treatment and prognosis of angiosarcoma of the scalp and face: a retrospective analysis of 48 patients. *The British Journal of Radiology*.

[6] Young RJ, Brown NJ, Reed MW, Hughes D, Woll PJ (2010). Angiosarcoma. *The Lancet Oncology*.

[7] Mentzel T, Kutzner H, Wollina U (1998). Cutaneous angiosarcoma of the face: clinicopathologic and immunohistochemical study of a case resembling rosacea clinically. *Journal of the American Academy of Dermatology*.

[8] Shehan JM, Ahmed I (2006). Angiosarcoma arising in a lymphedematous abdominal pannus with histologic features reminiscent of Kaposi’s sarcoma: report of a case and review of the literature. *International Journal of Dermatology*.

[9] Knight TE, Robinson HM, Sina B (1980). Angiosarcoma (angioendothelioma) of the scalp. An unusual case of scarring alopecia. *Archives of Dermatology*.

[10] Pan Z, Albertson D, Bhuller A, Wang B, Shehan JM, Sarma DP (2008). Angiosarcoma of the scalp mimicking a sebaceous cyst. *Dermatology Online Journal*.

[11] Aguila LI, Sánchez JL (2003). Angiosarcoma of the face resembling rhinophyma. *Journal of the American Academy of Dermatology*.

[12] Köhler HF, Neves RI, Brechtbühl ER, Granja NVM, Ikeda MK, Kowalski LP (2008). Cutaneous angiosarcoma of the head and neck: report of 23 cases from a single institution. *Otolaryngology—Head and Neck Surgery*.

[13] Holden CA, Spittle MF, Jones EW (1987). Angiosarcoma of the face and scalp, prognosis and treatment. *Cancer*.

[14] Pawlik TM, Paulino AF, McGinn CJ (2003). Cutaneous angiosarcoma of the scalp: a multidisciplinary approach. *Cancer*.

[15] Kitagawa M, Tanaka I, Takemura T, Matsubara O, Kasuga T (1987). Angiosarcoma of the scalp: report of two cases with fatal pulmonary complications and a review of Japanese autopsy registry data. *Virchows Archiv*.

[16] Nomura M, Nakaya Y, Saito K (1994). Hemopneumothorax secondary to multiple cavitary metastasis in angiosarcoma of the scalp. *Respiration*.

[17] Naka N, Ohsawa M, Tomita Y (1998). Prognostic factors in angiosarcoma: a multivariate analysis of 55 cases. *Journal of Surgical Oncology*.

[18] Mark RJ, Poen JC, Tran LM, Fu YS, Juillard GF (1996). Angiosarcoma: a report of 67 patients and a review of the literature. *Cancer*.

[19] Kacker A, Antonescu CR, Shaha AR (1999). Multifocal angiosarcoma of the scalp: a case report and review of the literature. *Ear, Nose and Throat Journal*.

[20] Lydiatt WM, Shaha AR, Shah JP (1994). Angiosarcoma of the head and neck. *The American Journal of Surgery*.

[21] Morrison WH, Byers RM, Garden AS, Evans HL, Ang KK, Peters LJ (1995). Cutaneous angiosarcoma of the head and neck. A therapeutic dilemma. *Cancer*.

[22] Ward JR, Feigenberg SJ, Mendenhall NP, Marcus RB, Mendenhall WM (2003). Radiation therapy for angiosarcoma. *Head and Neck*.

[23] Romanyshyn J, Wolden S, Caria N (2010). Radiation therapy in the treatment of angiosarcoma of the head and neck. *International Journal of Radiation Oncology Biology Physics*.

[24] Glickstein J, Sebelik ME, Lu Q (2006). Cutaneous angiosarcoma of the head and neck: a case presentation and review of the literature. *Ear, Nose and Throat Journal*.

[25] Patel VB, Speer TW (2012). Successful treatment of an angiosarcoma of the nose with radiation therapy. *Case Reports in Oncology*.

[26] Gkalpakiotis S, Arenberger P, Vohradnikova O, Arenbergerova M (2008). Successful radiotherapy of facial angiosarcoma. *International Journal of Dermatology*.

[27] Penel N, Lansiaux A, Adenis A (2007). Angiosarcomas and taxanes. *Current Treatment Options in Oncology*.

[28] Penel N, Bui BN, Bay J (2008). Phase II trial of weekly paclitaxel for unresectable angiosarcoma: the ANGIOTAX study. *Journal of Clinical Oncology*.

[29] Agulnik M, Okuno S, Von Mehren M (2009). An open-label multicenter phase II study of bevacizumab for the treatment of angiosarcoma. *Journal of Clinical Oncology*.

[30] George S, Merriam P, Maki RG (2009). Multicenter phase II trial of sunitinib in the treatment of nongastrointestinal stromal tumor sarcomas. *Journal of Clinical Oncology*.

[31] Maki RG, D’Adamo DR, Keohan ML (2009). Phase II study of sorafenib in patients with metastatic or recurrent sarcomas. *Journal of Clinical Oncology*.

[32] Park MS, Ravi V, Araujo DM (2010). Inhibiting the VEGF-VEGFR pathway in angiosarcoma, epithelioid hemangioendothelioma, and hemangiopericytoma/solitary fibrous tumor. *Current Opinion in Oncology*.

[33] Thong PS, Ong KW, Goh NS (2007). Photodynamic-therapy-activated immune response against distant untreated tumours in recurrent angiosarcoma. *The Lancet Oncology*.

[34] Jin I, Yuji M, Yoshinori N, Makoto K, Mikio M (2008). Anti-tumor effect of PDT using Photofrin in a mouse angiosarcoma model. *Archives of Dermatological Research*.

